# Hyperreactive malarial splenomegaly syndrome: a rare cause of splenomegaly in Switzerland (case report)

**DOI:** 10.1128/asmcr.00019-25

**Published:** 2025-04-03

**Authors:** Amelie Krug, Pablo Valladares, Amel Filali

**Affiliations:** 1 Division of Pediatrics, Department of Woman-Mother-Child, Lausanne University Hospital (CHUV) 527885, Lausanne, Switzerland; 2 Polyclinic of Tropical Medicine, Travel and Vaccinations, Center for Primary Care and Public Health (Unisante) 569258 , Lausanne, Switzerland; Rush University Medical Center, Chicago, Illinois, USA

**Keywords:** case report, hyperreactive malarial splenomegaly, malaria

## Abstract

**Background:**

Hyperreactive malarial splenomegaly syndrome (HMSS) is a rare cause of splenomegaly in a nontropical setting. Symptoms of HMSS are nonspecific, rendering the diagnosis challenging. If left untreated, the condition is potentially fatal.

**Case Summary:**

We herein detail the case of a young male presenting to the emergency department with fever and abdominal pain. Travel history included multiple trips to malaria endemic regions, the last trip dating back to 3 years before the consultation. Splenomegaly was identified via computed tomography (CT) scan, and a blood smear revealed *Plasmodium falciparum* infection (parasitemia of <0.1%). After excluding other causes of splenomegaly, HMSS was diagnosed and treated accordingly.

**Conclusion:**

A remarkable aspect of this case is the length of time elapsed between the last visit to an endemic region and symptom onset. This case report highlights the need to include HMSS in the differential diagnosis for splenomegaly in a traveler returning from an endemic region.

## INTRODUCTION

Hyperreactive malarial splenomegaly syndrome (HMSS) is a potentially life-threatening condition caused by an exaggerated immune response to recurrent or persistent malarial infections. HMSS frequently causes massive splenomegaly in countries burdened by malaria (1). Prevalence is challenging to estimate, given the paucity of literature on the subject. In Gambia, one study reports a rate of affected people of 1.6 per thousand people aged 10 years or older, whereas in the Upper Watut Valley of Papua New Guinea, a study reports that up to 80% of the adult population has HMSS (2, 3). Although rarely seen in Europe, this syndrome should be considered in patients with chronic splenomegaly returning from endemic regions. HMSS commonly affects young adults but can impact any age group (1). Disease occurs due to repeated or chronic immune stimulation by *Plasmodium* parasites, usually *P. falciparum*, but cases involving *P. vivax* or *P. malariae* are also documented (4, 5). Affected patients thus have a history of prolonged or repeated stays in malaria endemic regions (1, 4). It was discovered that genetics influence disease susceptibility, with the identification of HLA-DR2 as a genetic risk factor (6). Pathophysiology includes several steps. Initially, *Plasmodium*-specific and non-specific B-lymphocyte activation leads to abundant production of anti-*Plasmodium* antibodies and polyclonal IgM antibodies (4, 7). Researchers identified a deficit in CD8+ suppressor T-lymphocytes in patients with HMSS (8). The lack of B-cell inhibition by CD8+ contributes to the excessive antibody production. This hyper-IgM state precedes splenomegaly (4). Elevated levels of IgM lead to the formation of immune complexes, inducing cold-agglutinin hemolysis (1, 4). Persistent splenic filtration of immune complexes and erythrocytes leads to reticulo-endothelial hyperplasia (9). Concomitantly, chronic *Plasmodium* infection leads to loss of adhesins on the surface of infected erythrocytes, causing sequestration and thus splenomegaly (10). Clinical presentation of HMSS includes massive splenomegaly and non-specific symptoms, like low-grade fever, fatigue, and abdominal pain. Patients remain paucisymptomatic for extended periods of time, and symptoms may only become apparent months after leaving an endemic region (1). Diagnosis relies on the Fakunle criteria (Table 1), last amended by Bates et al. in 1997 (11). The diagnosis typically requires fulfillment of all the major criteria. HMSS carries significant morbidity and mortality. Hypersplenic-associated cytopenias are common (4, 9, 12). Anemia is frequent, and its etiology is multifaceted, including splenic sequestration and immunological hemolysis due to cold agglutinins (4). Leukopenia and thrombocytopenia are thought to be the result of splenic sequestration. HMSS is considered a premalignant state, which, if untreated, may progress to splenic lymphoma (4, 7). Other causes of mortality include splenic rupture and infection. Rates of these complications remain unknown. A 1972 study in Papua New Guinea estimated an overall mortality rate of 57% for untreated patients with Grade V splenomegaly (Hackett’s spleen size classification) (13). Mortality is lower in high-income countries, with no deaths reported in the three European case series concerning HMSS in migrants and returning travelers (14–16). Treatment regimens vary between different regions of the world (1). Treatment typically consists of prolonged or repeated courses of antimalarial medications in endemic regions. In regions free from parasitic reexposure, treatment is effective with a single antimalarial course (1, 14–16).

**TABLE 1:** Fakunle’s criteria, as modified by Bates and Bedu-Addo (1)

Major criteria	Minor criteria
Gross splenomegaly: 10 cm or more below the costal margin in adults, without identifiable origin	Hepatic sinusoidal lymphocytosis
Elevated serum IgM level (>2 standard deviations or more above the local mean)	Normal cellular and humoral responses to antigenic challenge, including phytohemagglutinin stimulation
Favorable clinical and immunologic responses to antimalarial therapy	Hypersplenism
Elevated antibody levels of *Plasmodium* species (≥1:800)	Lymphocytic proliferation
	Occurrence within families or tribes

## CASE PRESENTATION

**Fig 1:**
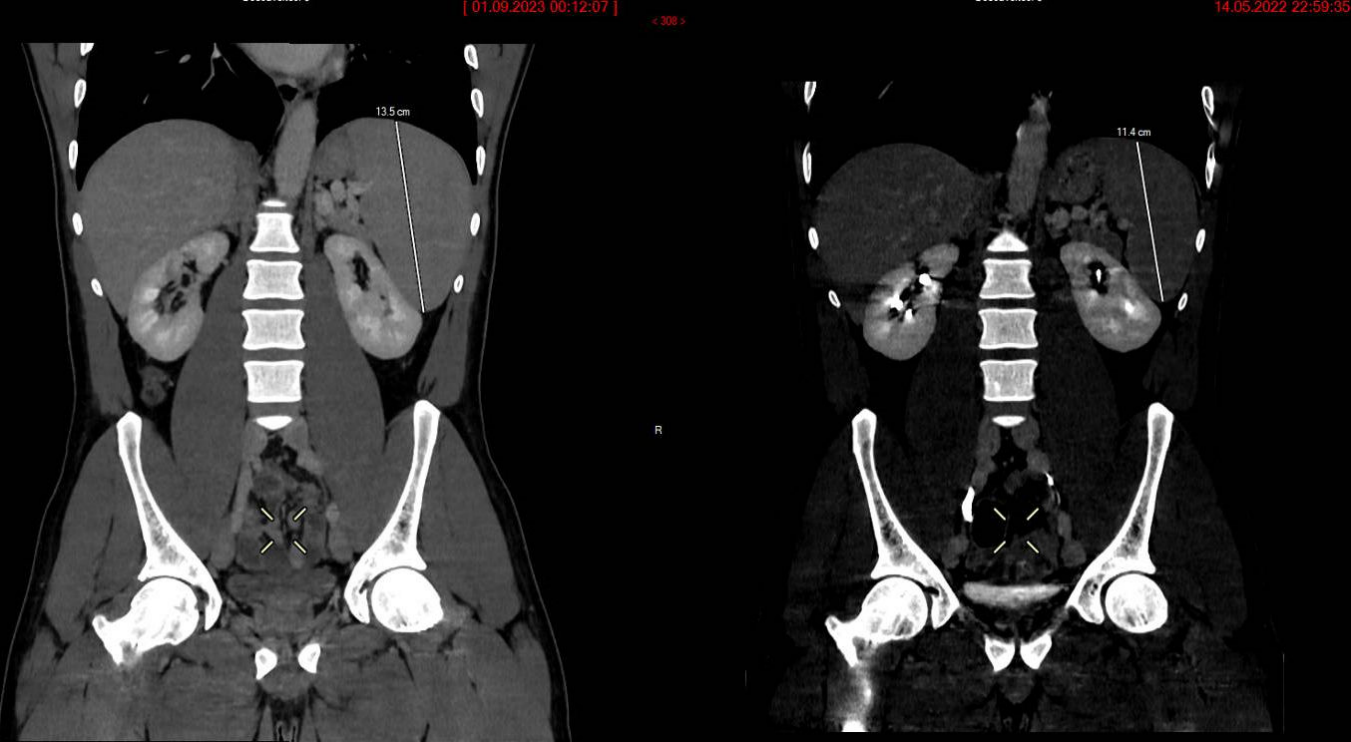
Abdominal CT scans showing worsening splenomegaly. The first scan (right) shows splenic dimensions of 11.4 × 12 × 5 cm. The second scan (left), 7 months later, shows increased spleen dimensions of 13.5 × 13 × 7 cm.

We herein detail the case of a young male from Sub-Saharan Africa presenting to the emergency department in a Swiss hospital with fever and periumbilical abdominal pain. Abdominal palpation was unremarkable. Blood work revealed leukopenia (3.2 g/L) and thrombocytopenia (55 g/L), with elevated inflammatory markers (CRP 111 g/L). The patient had a history of splenomegaly of unidentified origin, diagnosed on an abdominal computed tomography (CT) scan after he had presented to the emergency department with similar complaints 1 year prior. A new CT scan (Fig. 1) showed worsening splenomegaly (from 11.4 × 12 × 5 cm to 13.5 × 13 × 7 cm) in comparison to the previous scan. Medical history was notable for several trips to malaria-endemic regions, with prolonged stays, over the previous decade. The last trip was 3 years before presenting to the emergency department. The patient stated that malaria prophylaxis (doxycycline) was taken on these trips, however, not consistently. A blood smear revealed *P. falciparum* infection with a parasitemia of <0.1% and positive *Plasmodium* antibodies (immunofluorescence antibody test titer, 1:1,280). IgM antibody levels were not obtained as the patient was subsequently lost to follow-up. The patient was diagnosed with HMSS when other potential etiologies for splenomegaly were excluded, and he was treated with artemether/lumefantrine at standard regimen. He was referred for an outpatient consultation in the tropical medicine clinic to which he never presented himself. Clinical evolution after treatment remains unknown due to lack of follow-up.

## DISCUSSION

This case report adds to the scarce literature on the subject of HMSS, concerning travelers returning from a malaria endemic region. To date, the majority of literature on HMSS is limited to case reports and case series. In 2015, Leoni et al. published the only existing systematic review on the subject, regrouping 89 articles, in which they noticed an “extreme poverty of papers dealing with HMSS” (1). Much remains unknown on the topic, notably in terms of disease prevalence and rates of complications, as well as how HMSS affects the pediatric population. HMSS is difficult to diagnose, in part because of the lack of specific symptomatology. In a study of 49 patients with hyperreactive malaria (with or without splenomegaly) returning to Belgium from malaria-endemic regions, fever was present in a minority (39%) of patients, whereas 57% presented with fatigue and 45% with weight loss (15). Similar results were reported in an Italian study of 44 patients with HMSS by Bisoffi et al. (16). Our patient presented to the emergency department solely with fever and nonspecific abdominal pain. These symptoms potentially correlate with many different infectious, oncological, or inflammatory diseases. Among healthcare workers, there is a general lack of awareness of this form of chronic malaria, particularly in non-endemic regions of the world. This may lead to misdiagnosis or underdiagnosis, which likely happened to this patient when he presented to the emergency department the first time. In the case of chronic splenomegaly with a history of travel to a malaria-endemic region, it is important to consider HMSS in the differential diagnoses to treat this potentially fatal condition. An unusual aspect to this case is the length of time elapsed between the last visit to an endemic region. To date, there are three European case series on HMSS in migrants or returning travelers. Two of these (Puente et al. and Van den Ende et al.), cite the length of time between arrival in the respective countries and the first consultation to a clinic where HMS was diagnosed, with a median of 11 days and 21 days respectively, but no more than 1 and 3 months for each (14, 15). For our patient, *P. falciparum* PCR detection was positive 3 years after returning from an endemic region, which is longer than in most case reports. This case report presents several limitations. Diagnosis was made based on the presence of splenomegaly upon abdominal CT, with positive *P. falciparum* antibody titer (IFAT 1:1280), positive PCR test, and the presence of low-grade *P. falciparum* parasitemia (<0.1%), with a history of prolonged stays in malaria-endemic regions and the absence of an alternative explanation for the splenomegaly. IgM antibody levels were not obtained, and treatment response was not documented since the patient was lost to follow-up. Two out of four of Fakunle’s major criteria for HMSS diagnosis were thus met (Table 1). In their systematic review, Leoni et *al*. report that cases that did not fully fit Fakunle’s criteria, and were therefore untreated, progressed to HMSS. They thus recommend the treatment of incomplete or “early” HMSS (1). The Fakunle criteria were last revised in 1997 (1). Throughout the literature, many authors do not strictly adhere to these criteria to make the diagnosis of HMSS. Leoni et al. reported five studies where patients diagnosed with HMSS did not have raised IgM levels, and two studies where authors did not deem this a necessary criterion for diagnosis (1). Another highly variable factor in the diagnostic criteria is the method for measuring spleen size, with some authors using Hackett’s classification and others relying on imaging (mainly ultrasound) (1, 14–16). Our patient’s spleen was not palpable, but splenomegaly was detected via CT scan. There is a call among authors to update the diagnostic criteria in light of biomedical evolution. McGregor et al. suggested the inclusion of PCR as a diagnostic criterion, after showing that patients with positive PCR results responded well to treatment, and those who were negative did not (7). PCR also permits identification of the *Plasmodium* species and detection of *Plasmodium* despite low parasitemia, which is usually the case in chronic infection (9).

### Conclusion

HMSS is a rare cause of splenomegaly outside of malaria-endemic regions but should be considered in patients who have a history of prolonged or repeated stays in malaria-endemic regions, even if these stays were many years prior. This case adds to the scarce literature on the topic of HMSS and raises questions concerning the need to update the criteria used for diagnosis of this condition. It highlights the need for further studies on the epidemiology and complications of HMSS, particularly given the elevated estimated incidence of the condition in malaria-burdened regions of the world.
